# Computational design of fusion proteins against ErbB2-amplified tumors inspired by ricin toxin

**DOI:** 10.3389/fmolb.2023.1098365

**Published:** 2023-03-02

**Authors:** Yasser Ahmadi Moghaddam, Asad Maroufi, Sara Zareei, Mehdi Irani

**Affiliations:** ^1^ Department of Plant Production and Genetics, Faculty of Agriculture, University of Kurdistan, Sanandaj, Iran; ^2^ Department of Cell & Molecular Biology, Faculty of Biological Sciences, Kharazmi University, Tehran, Iran; ^3^ Department of Chemistry, Faculty of Science, University of Kurdistan, Sanandaj, Iran

**Keywords:** chimeric protein, cancer, ErbB2, ricin, molecular dynamics

## Abstract

Although the anti-cancer activity of ricin is well-known, its non-specific targeting challenges the development of ricin-derived medicines. In the present study, novel potential ribosome-inactivating fusion proteins (RIPs) were computationally engineered by incorporation of an ErbB2-dependant penetrating peptide (KCCYSL, MARAKE, WYSWLL, MARSGL, MSRTMS, and WYAWML), a linker (either EAAAK or GGGGS) and chain A of ricin which is responsible for the ribosome inactivation. Molecular dynamics simulations assisted in making sure that the least change is made in conformation and dynamic behavior of ricin chain A in selected chimeric protein (CP). Moreover, the potential affinity of the selected CPs against the ligand-uptaking ErbB2 domain was explored by molecular docking. The results showed that two CPs (CP2 and 10) could bind the receptor with the greatest affinity.

## 1 Introduction

Ribosome-inactivating proteins (RIPs) are a family of N-glycosidases that inhibit eukaryotic protein translation irreversibly (Wong et al., 2020) by removing the adenine A4324 base of 28 S ribosomal rRNA and subsequently avoiding the interaction between 60s subunit and elongation factor (eEF-1) (Stirpe and Battelli, 2006). RIPs produce immunity against pathogens such as fungi, bacteria, viruses, and insects (Stevens et al., 1981; Wang and Turner, 2000; Akkouh et al., 2015; Wong et al., 2020) for the plants, fungi, and algae from which they originated. In medicine, RIPs are considered promising anti-fungal (Landi et al., 2022), anti-viral (Citores et al., 2021), and anti-tumor (Virgilio et al., 2010) agents, and their beneficial effects are shown in several types of cancer, such as breast cancer (Fang et al., 2012), lymphoma (Wang et al., 2007), and colon cancer (Huang et al., 2010). Moreover, there are attempts to enhance the pharmacological activity of RIPs (Lu et al., 2020).

**FIGURE 1:**
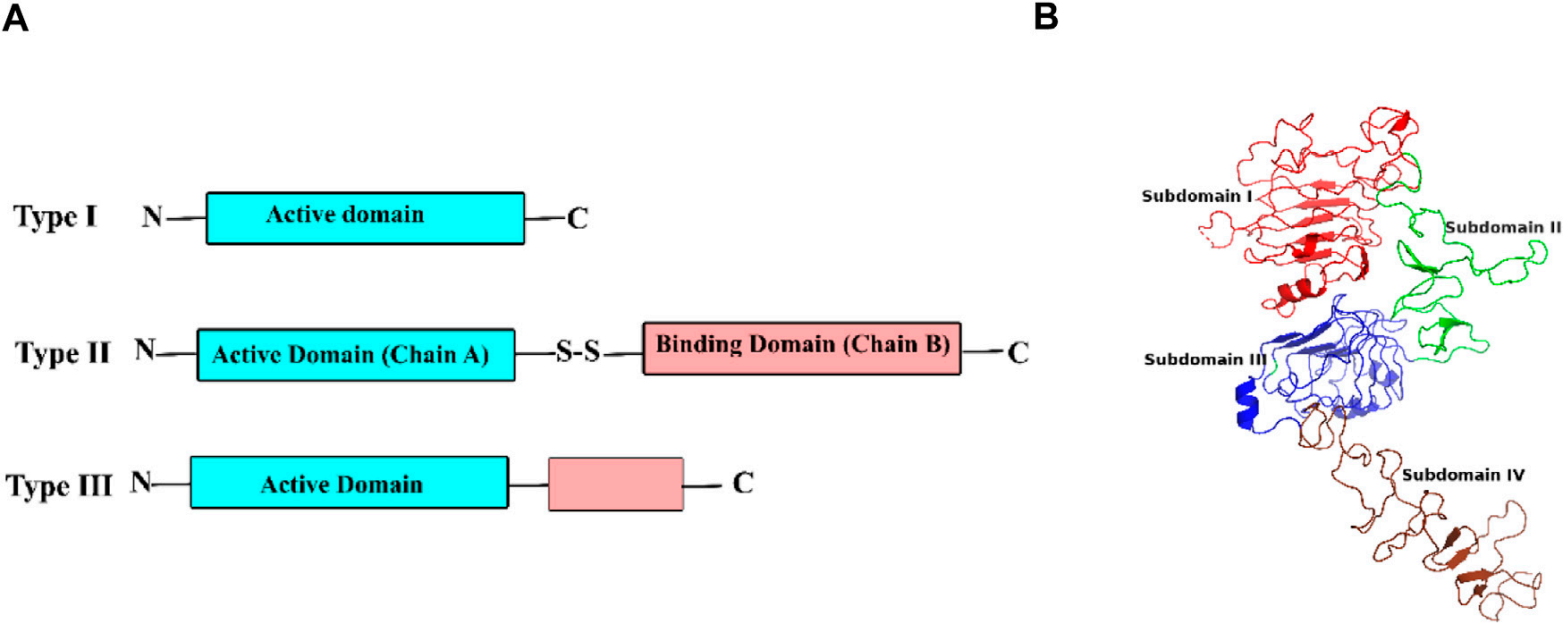
**(A)** The structure of three types of RIPs (ribosome inactivating proteins) and **(B)** extracellular domains of ErbB2 (PDB ID 6OGE).

RIPs can be classified into three main types based on their structures. Type I is a monomer, whereas types II and III include two chains. Type II RIPs consist of chain A which has anti-translation activity and chain B which is responsible for binding to the cell surface. These domains are connected by a disulfide bond (Figure 1A). The attachment of Chain B to galactose-terminated surface glycoproteins and glycolipids endows type II RIPs with the pharmaceutical benefit of cell penetration by endocytosis in a clathrin-dependent- or independent manner (Barbieri et al., 1993). However, this has brought a challenge for the development of type II-derived drugs against tumor cells because chain B is not capable of specific recognition of a cell of a specific type and thus targets a wide range of cellular receptors. This property underpins the toxicity of type II RIPs against almost any cell line (Fredriksson et al., 2015).

Ricin is a type II RIP derived from the castor bean (*Ricinus communis* L.) which is known as a promising anti-tumor agent (Mosinger, 1951) due to its high ribosome inactivating rate (Eiklid et al., 1980). However, ricin also suffers from the non-specific recognition of many cell types (Audi et al., 2005; Abbes et al., 2021). To combat this issue, some strategies such as chemical modification, inhibition, or removal of chain B have been proposed. Instances of the last option include the addition of an anti-tumor antibody (Kanellos et al., 1989; Masui et al., 1989; Li et al., 2016), liposome (Tyagi and Ghosh, 2011; Loan et al., 2019), nanocarrier (Nicolson and Poste, 1978), or fusion protein (O'Hare et al., 1990) to chain A in the absence of chain B.

Although some studies have eliminated chain B, a non-specific effect of ricin_A_ was seen (Krolick et al., 1980; Vitetta, 1988) indicating the importance of adding tumor-delivering agents to ricin_A_. Accordingly, there are many studies in which immunotoxins consisting of ricin_A_ and an antibody were proposed and designed against various cancer conditions (Ramakrishnan and Houston, 1984; Thiesen et al., 1987; Oratz et al., 1990; Schmidberger et al., 1990; Selvaggi et al., 1993). Peptide-Targeted Silica Nanoparticle-Supported Lipid Bilayers were also proposed for the delivery of ricin_A_ in hepatocellular carcinoma (Epler et al., 2012). Ricin_A_ was also conjugated with an ErbB2-targeting affibody and KDEL signal peptide. It was shown that this immunotoxin has higher toxicity against cancer cells compared to doxorubicin (Park et al., 2022).

Human epidermal growth factor receptor 2 (ErbB2, HER2, or neu) is overexpressed on the cellular surface of tumors (Roy et al., 2019; Işık and Barut, 2020; Egebjerg et al., 2021; Omranipour et al., 2021), especially in breast (Oh and Bang, 2020) and gasteric (Boku, 2014) cancers. From a structural perspective, ErbB2 is formed by three main domains: an extracellular domain consisting of four subdomains I, II, III, and IV, a membrane-embedded region, and an intercellular domain with tyrosine kinase activity (Cho et al., 2003) (Figure 1B). ErbB2 has a high extracellular accessibility and can internalize and uptake its ligands into the cell. This makes it an ideal delivery vehicle for anticancer agents like nanoparticles (Wartlick et al., 2004) and antibodies (Leyton, 2020). A well-established example is trastuzumab which is shown to penetrate cells through endocytosis of the ErbB2 domain IV (Austin et al., 2004; Ren et al., 2012). Therefore, the trastuzumab-binding pocket can be considered a region that has the potential for ligand endocytosis.

In the present study, we aimed to rationally design safe anti-cancer chimeric proteins against ErbB2-positive tumor cells through computational approaches. Chimeras contained Ricin_A_ due its inhibitory potential against protein translation. To increase the specificity of CPs, peptides with approved affinity for the trastuzumab-binding pocket of ErbB2 were added to ricin_A_ with the assistance of either a rigid or flexible linker. In addition, we showed that the least change is made in conformation and dynamic behavior of ricin_A_ moiety in selected chimeric proteins. Also, the potential affinity of the selected CPs against the ligand-uptaking domain of ErbB2 was explored. This means that the designed selected CPs are capable of being enternalized by ErbB2 into the cell where their ribosome inactiving activity can be of assistance for cancer therapy.

## 2 Computational methods

### 2.1 Chimeric proteins

**FIGURE 2:**
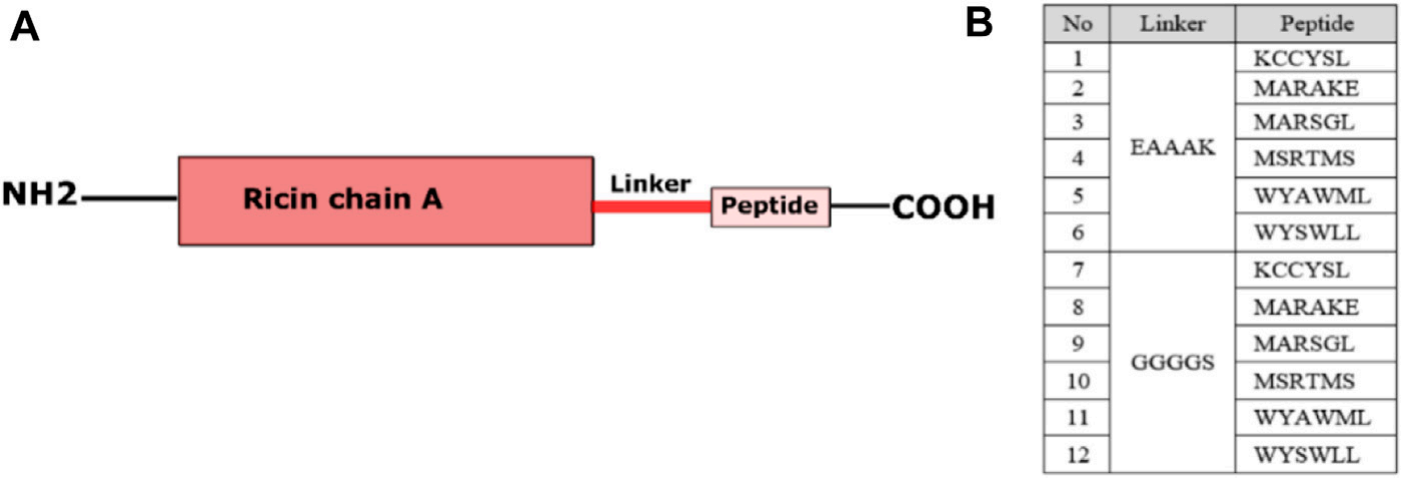
The overall view of the 12 designed chimeric protein moieties.

Chimeric proteins (CP) included ricin_A_, a linker, and an approved ErbB2-specific penetrating peptide (Figure 2A). First, the amino acid composition of ricin_A_ was retrieved from PDB ID 2AAI in FASTA format (Rutenber et al., 1991). Then, either a flexible (GGGGS) or rigid (EAAAK) linker, and a peptide (KCCYSL (Ringhieri et al., 2017), MARAKE (Houimel et al., 2001), WYSWLL (Karasseva et al., 2002), MARSGL (Sugo et al., 2013), MSRTMS (Houimel et al., 2001), and WYAWML (Karasseva et al., 2002)) were added to the end of ricin_A_ sequence manually in a text editor (Figure 2B). The peptide sequences were obtained from the Immunet BDB database (http://immunet.cn/bdb).

### 2.2 Homology modeling and quality assessment

3D structures of all templates were predicted by the MODELLER software 15.9 (Eswar et al., 2006) based on the most similar available conformations. The best final models were selected after their qualities were checked by the ERRAT (Colovos and Yeates, 1993) and VERIFY3D servers. Moreover, Ramachandran diagrams were obtained using the PROCHECK server (Laskowski et al., 1993) (Supplementary Figure S3).

### 2.3 Molecular dynamics and docking

Molecular dynamics (MD) simulations were employed to investigate whether the ricin_A_ moiety of CPs retains the conformation it had in ricin since any significant change in ricin_A_ may affect the ribosome-inactivating activity of CPs. MD simulations were performed using the GROMACS package, version 2020 (Van Der Spoel et al., 2005), and Charmm36 forcefield for making topology files. All systems were solvated in a cubic box with a minimum distance of 1.0 nm from the edges of the box and filled with SPC water molecules (Mark and Nilsson, 2001) as a reliable water model for aqueous solutions of biomolecules (Zielkiewicz, 2005).

Energies of all systems were minimized for 50,000 steps, followed by a thermal equilibrium step using the Berendsen thermostat at 310 K. Finally, systems were subjected to 100-ns production simulations. The MD trajectories were saved every 10 ps. The pressure was equilibrated for 1 ns to achieve the pressure of 1 bar using the Berendsen barostat. LINCS (Hess et al., 1997) and PME mesh (Darden et al., 1993) algorithms were used to constrain the bond parameters and the calculation of long-range electrostatic interactions, respectively. During the simulations, the Fourier grid spacing and Coulomb radius were set at 0.16 and 1.2 nm, respectively. The cutoff radius for van der Waals interactions was 1.2 nm.

It is proved that a protein’s activity depends on its dynamic behavior (Daniel et al., 2003; Torgeson et al., 2022). Therefore, CPs whose ricin_A_ moieties showed the greatest similarity in behavior during the MD simulations to that of chain A were considered to have a minimal change in their ribosome-inactivating activity and were subjected to molecular docking simulations against ErbB2. Docking was carried out by the HADDOCK server (Dominguez et al., 2003) which is a fully automated server designed for protein-protein docking simulations. It requires PDB files of the proteins as input. The docking score of this server is a linear sum of energy terms such as van der Waals, electrostatic, desolvation, and restraint violation energies the higher quantities of which indicate greater energy constraints of complex formation.

To validate the docking studies, trastuzumab was docked against ErbB2. The X-ray crystallized PDB ID 6OGE (Hao et al., 2019) was obtained from the RCSB data bank. It included trastuzumab, Fab-Pertuzumab, and ErbB2. After making sure that HADDOCK produced the same interactions and orientations of trastuzumab, CPs were docked and the trastuzumab-binding region of ErbB2 was introduced (Hao et al., 2019) as the active residues to the server while the remained parameters remaining as default. Then, the complexes with the lowest binding scores were selected. Finally, we used PRODIGY server (Xue et al., 2016) to decipher the potential binding affinity of each CP for the receptor.

### 2.4 Toxicity assessment

The toxic potential of CPs was examined by the ToxDL server which is devised to predict toxic domains in protein structures by deep learning (http://www.csbio.sjtu.edu.cn/bioinf/ToxDL/) (Pan et al., 2021).

## 3 Results and discussion

This study seeks to rationally design novel chimeric proteins against ErbB2-positive cells by disturbing their protein synthesis. Most CPs suppressing ErbB-2 activity in tumor cells are antibody-based inhibitors. For instance, Erb-38 is a chimeric protein, which showed its beneficial activity in breast cancer (Reiter et al., 1994). It is made of Mab23, the dsFv fragment of an anti-ErbB2 antibody, and *Pseudomonas* exotoxin (PE38). Here, we exploited the toxic potential of ricin_A_ and ErbB-2-dependant penetrating peptides to limit the non-specific targeting of ricin.

### 3.1 Chimeric proteins

Six peptides with the capability of binding to the trastuzumab-binding pocket were connected to ricin_A_ by either a rigid or a flexible linker (Figure 2) which resulted in 12 initial templates. The rigid linker (EAAAK) is an α-helix maker previously used to design fusion proteins against various cancers such as breast (Thiesen et al., 1987; Epler et al., 2012) and cervical (Park et al., 2022) cancer. It contains a salt bridge between its glutamic acid and lysine which keeps a fixed distance between the peptide and ricin_A_, leading to the maintenance of independent functions of these compartments by creating a stable helix structure (Zielkiewicz, 2005).

The flexible linker included smaller GGGGS residues (Klein et al., 2014). Flexible linkers are usually applied when the connected domains require a certain degree of movement or interaction. This linker has been designed for recombinant fusion proteins and is used to treat some cancers (Valiyari et al., 2020).

Two main considerations of designing proteins are their foldability and functionality which in most cases are related to each other. Here, although the length of peptides and linkers are shorter compared with ricin_A_, it is not unlikely they can disturb the foldability of CPs and hence negatively impact their ribosome inactivating function. To elucidate any potential effect of adding such sequences, we used homology modelling and molecular dynamics simulations.

### 3.2 Homology modeling and quality assessment

To elucidate the 3D conformations, all of twelve initial templates were subjected to homology modeling. Models with the best dope scores (the lowest scores) were selected as final 3D structures. Moreover, the quality of models was determined by three programs ERRAT (Colovos and Yeates, 1993), VERIFY3D (Eisenberg et al., 1997), and PROCHECK (Laskowski et al., 2006) on the SAVES server (Tables 1, 2; Supplementary Figures S1, S2). ERRAT evaluates non-bonded atomic interactions, and its higher scores indicate higher quality. The generally accepted score of >50 indicates a high-quality model (Table 1). VERIFY3D determines the compatibility of an atomic model (3D) with its amino acid sequence. It assigns a structural class to each residue (alpha, beta, loop, polar, non-polar, etc.) and compares the results to other structures. VERIFY3D scores of higher than 80% means the acceptable quality of the model (Table 1). Ramachandran plots of the models (Supplementary Figure S3) were depicted, and their statistics are shown in Table 2. It can be seen that at least 83.7% of residues were in the most favorable regions and maximally 1.3% in disallowed regions, suggesting an acceptable quality of the models.

**TABLE 1:** Validation of the predicted CPs by Verify-3D and ERRAT scores.

Structure	Verify-3D (Averaged 3D-1DScore> =0.2) (%)	ERRAT score
Ricin_A_	—	80.695
1	95.32	74.157
2	92.81	76.962
3	93.53	68.165
4	94.60	72.285
5	92.81	79.623
6	94.24	69.582
7	98.92	79.468
8	100	70.787
9	96.76	73.764
10	100	78.571
11	95.32	75.285
12	94.96	69.962

**TABLE 2:** Validation of the predicted chimeric proteins based on their Ramachandran plots.

Structures	Residues in most favorable regions (%)	Residues in additional allowed regions (%)	Residues in generously allowed regions (%)	Residues in disallowed regions (%)
Ricin_A_	83.7	12.4	2.6	1.3
1	88.5	8.6	1.6	1.2
2	91.4	7.0	0.8	0.8
3	90.1	7.8	1.2	0.8
4	90.6	7.0	1.6	0.8
5	90.6	7.8	0.8	0.8
6	90.6	6.6	2.0	0.8
7	90.4	7.1	1.2	1.2
8	90.4	5.8	2.9	0.8
9	90.4	7.1	1.7	0.8
10	90.8	7.1	1.2	0.8
11	89.6	6.7	2.9	0.8
12	91.7	5.8	1.7	0.8

The quality of the homology model results indicates that the addition of linkers and peptide sequences had no negative effect on foldability of ricin_A_.

### 3.3 MD results

To make sure that the integrity, structure, and subsequent inhibitory activity of ricin_A_ moiety is not affected by the addition of linkers and peptides, we performed MD simulations. CPs whose ricinA moiety’s behavior was the most similar to that of free ricinA were then subjected to molecular docking.

**FIGURE 3:**
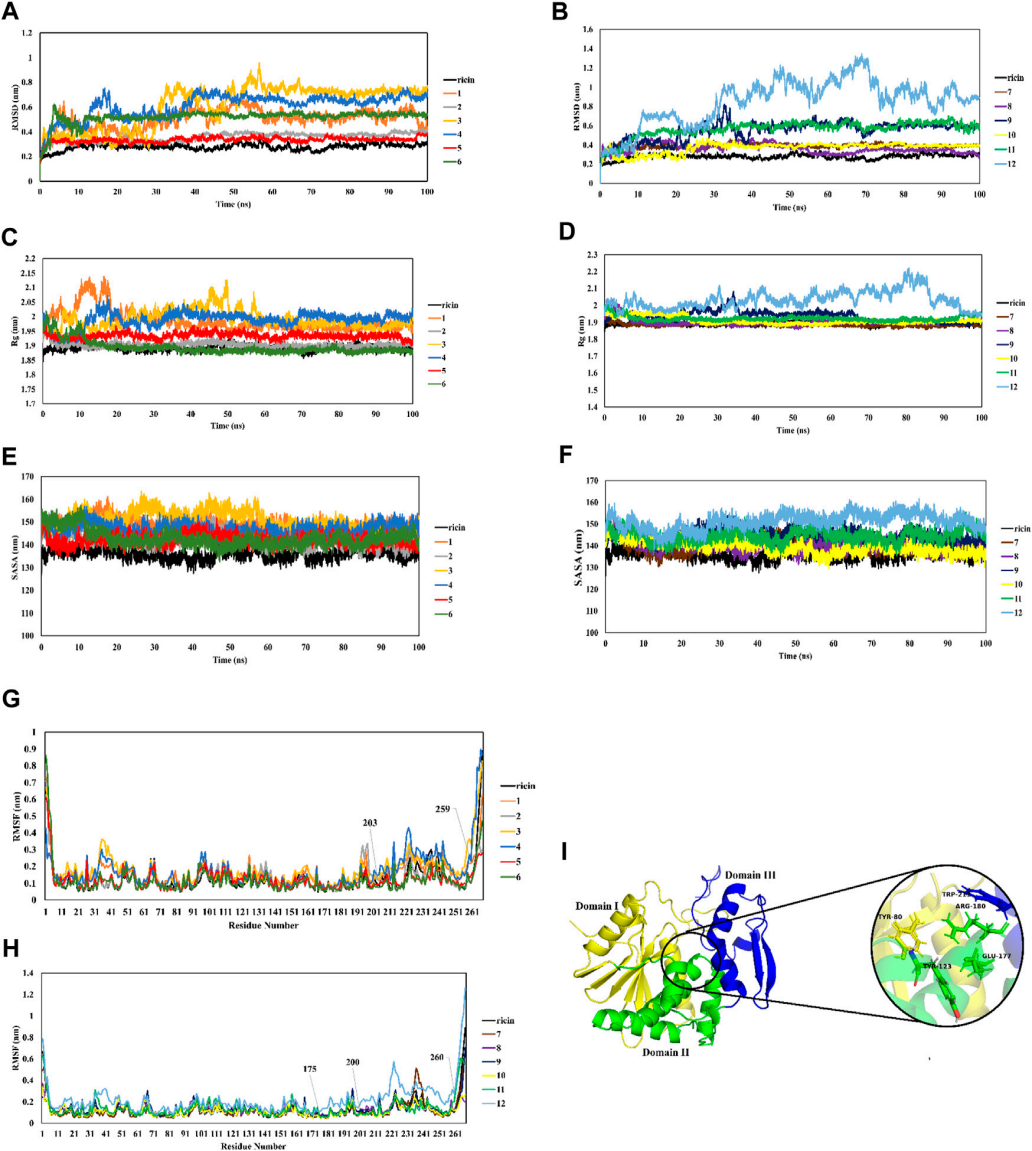
MD analysis of dynamic behavior of CPs in comparison with ricin. RMSD **(A,B)**, Rg **(C,D)**, SASA **(E,F)**, RMSF **(G,H)**, and structure view of ricin_A_ **(I)**.

**FIGURE 4:**
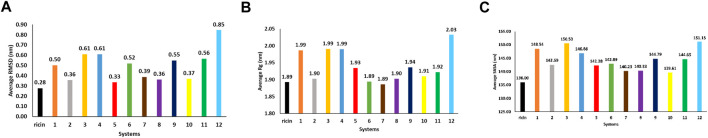
plots of average RMSD **(A)**, Rg **(B)**, and SASA **(C)** of all systems during 100-ns simulations.

The RMSD of ricin’s backbone atoms compared to the initial structure as a reference is shown in Figures 3A, B, and their average values are shown in Figure 4A. In the CP12 structure, ricin_A_ has the least stable structure as seen from the severe fluctuations in its plot. The ricin_A_ moiety of CP12 also showed the greatest divergence from ricin_A_ in ricin compared to other CPs, with an average RMSD of 0.8 (Figure 4A). Moreover, except for CPs 2, 5, 7, 8, and 10, which had the closest plots and average values (0.35, 0.33, 0.38, 0.36, and 0.36 nm, respectively) to ricin, other CPs diverged from ricin dramatically, suggesting a greater structural change in ricin_A_ moiety when it is joint with peptides and linkers in these CPs.

To further explore ricin’s significant structural changes in each CP, further analyzes were carried out. It is shown in Figures 3C, D that ricinA‘s radius of gyration (Rg) in CPs 1, 3, 4, and 12 (with the average values of 1.99 nm for CPs 1, 3, and 4 and 2.03 nm for CP12) diverged the most from that of ricin (with the average Rg of 1.89 nm) (Figure 4B) suggesting that the addition of their relevant recombinant moiety may induce significant structural change in ricin_A_ structure. This matter increases the possibility of losing the ricin_A_ inhibitory effect in these CPs. On the other hand, the remaining CPs had favorable Rg values (Figure 4B).

SASA (solvent-accessible surface area) analysis is known as an indicator of the surface area of the protein. Increased values of SASA suggest the expansion of the protein structure. SASA plots of all systems are shown in Figures 3E, F and their average quantities are depicted in Figure 4C. Compared with free ricin (SASA = 136 nm), it can be seen that CPs 2, 5, 6, 7, 8, and 10 have the closest SASA values to free ricin. This suggests that the ricin moiety of these CPs has a similar surface to free ricin. The Rg and SASA plots show consistency so that CPs with a significant increase in ricin’s Rg exhibited a more divergent SASA than the free ricin.

Furthermore, RMSF analysis was performed to understand better the mobility of the ricin part in each CP. Figures 3G, H show that residues 175–260 in the ricin part of CPs 4, 7, and 12 have the most significant movements during the simulations. This suggests that adding a linker and peptide to ricin_A_ increased the flexibility of this region compared to single ricin. Ricin_A_ has three main regions: domain I consist of β -sheets, while domain II is an α -helical structure. Domain III plays a major role in dimer formation by binding to chain B. The active site of ricin contains highly conserved residues (Tyr80, Tyr123, Glu177, Trp211, and Arg180 (Eswar et al., 2006); see Figure 3I). As seen from the RMSF plots in Figure 3, the most fluctuating region of CPs 4, 7, and 12 is located within ricin’s active site, which can diminish the catalytic activity of these complexes. Therefore, this matter can be another reason for removing these CPs from our test cases.

**FIGURE 5:**
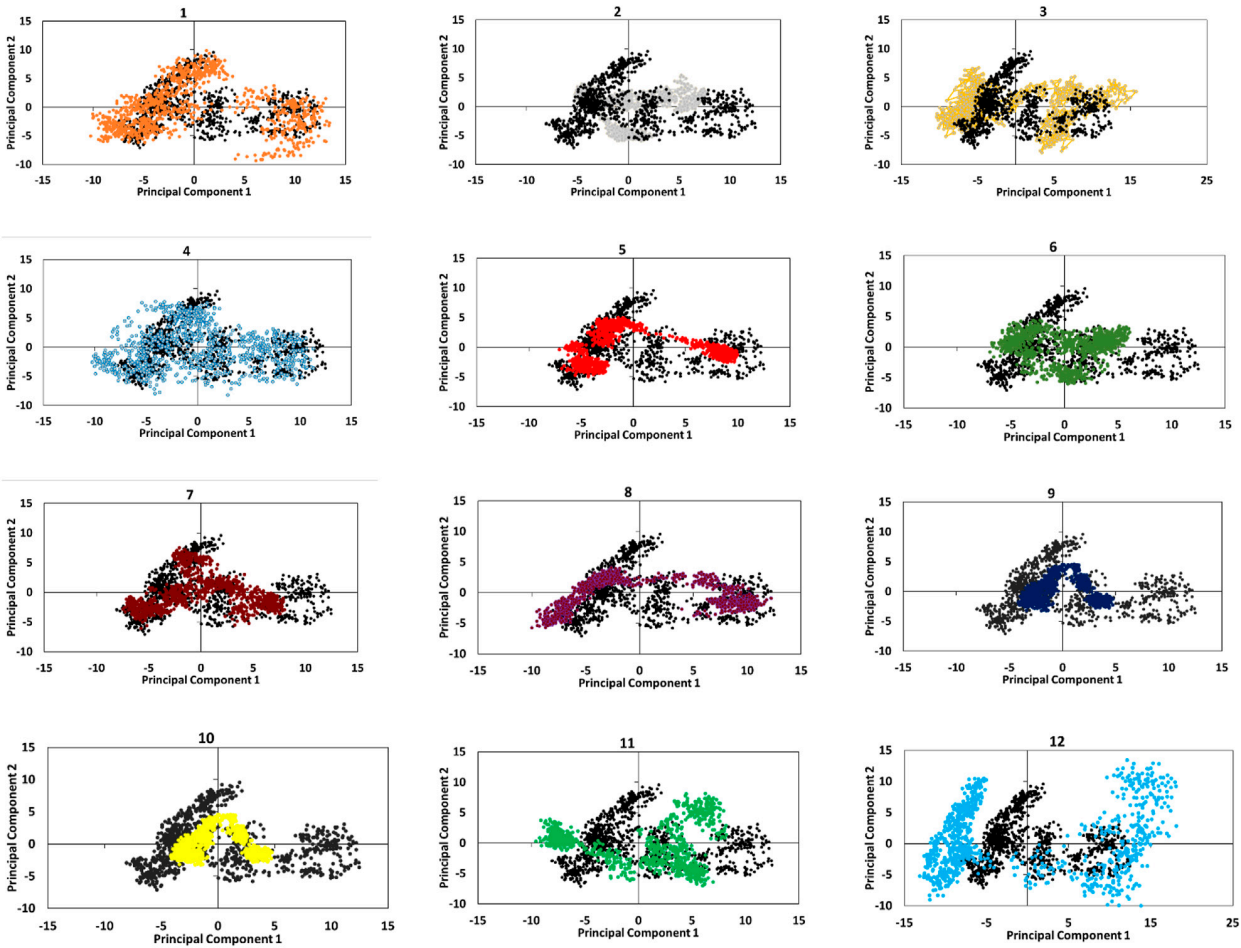
dynamic behaviors of CPs compared with single ricin (black) revealed by the projection of eigenvectors on the first two principal components.

Furthermore, principal component analysis (PCA) of Cα atoms of ricin was employed to understand how the protein backbone in CPs behaves during the simulations (Figure 5). The results show that CPs 1, 3, 4, 11, and 12 have greater overall motions than ricin_A_ since they cover a broader part of their conformational space. This implies that adding linkers and peptides increases collective motions in the ricin compartment, which in turn may affect ricin’s functionality. The remaining CPs that showed similar behaviors to ricin in the previous MD analyses had smaller conformational space coverage, indicating their limited collective motions. This suggests that adding the linker and peptides in these CPs makes ricin_A_ rather rigid.

According to the analyzes performed in the MD section (RMSD, RMSF, Rg, SASA), CP2, 5, 7, 8, and 10 had the most stable structure and were the most similar to free ricin_A_. Thus, we chose them for the docking step. It is worthy of note that none of the designed CPs had toxic potential according to the ToxDL server results (Supplementary Table S1). Keeping a balance between having toxicity for tumors and safety for normal cells might be a challenge in protein design. However, the proposed CPs make the common solutions like designing a carrier unnecessary because while they are safe, they may still have their ribosome inactivating potential. Another challenge that needs to be addressed is the specificity and internalization of CPs. Instead of using a carrier, we hypothesized that trastuzumab binding cavity of ErbB-2 can be exploited as a natural carrier for designed CPs. Moreover, this affords the opportunity of providing specificity for CPs. We examined this idea with molecular docking.

### 3.4 Docking analysis

**FIGURE 6:**
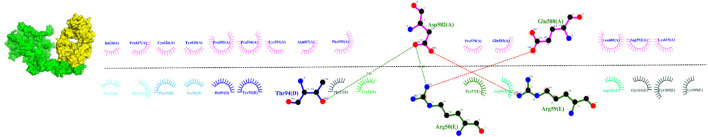
the interaction between trastuzumab (yellow, chain E) and ErbB2 (green, chain A) extracted from 6OGE PDB ID.

**FIGURE 7:**
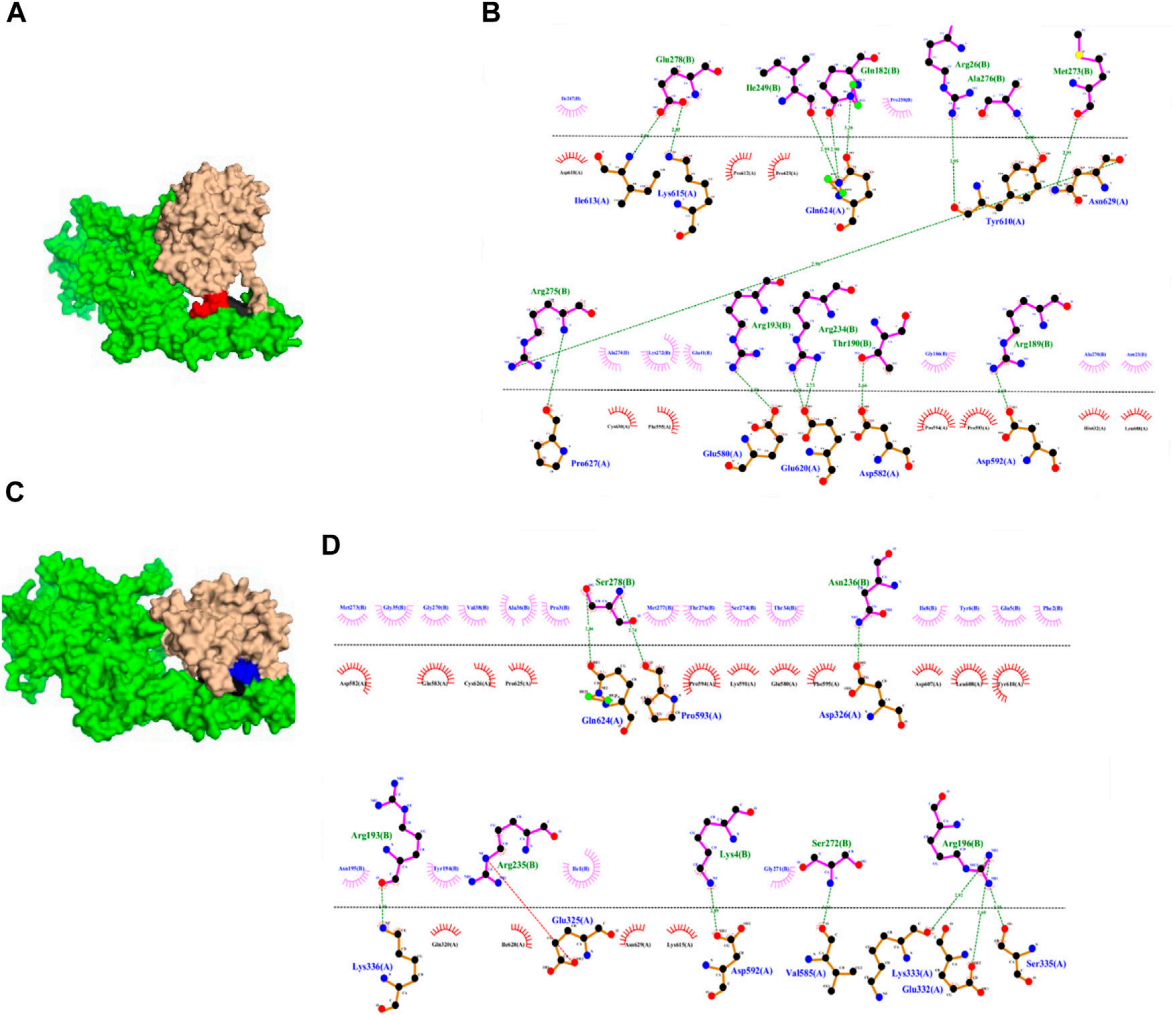
the interaction between CPs 2 **(A,B)** and 10 **(C,D)** with ErbB2 (green). Ricin_A_ is depicted in salmon, while linkers are shown in black. Peptides MARAKE, and MSRTMS are colored red and blue, respectively.

MD results indicated that ricin_A_ of CPs 2, 5, 7, 8, and 10 may most likely retain its inhibitory activity. Using molecular docking, we aimed to examine whether the selected CPs were capable of binding to trastuzumab-binding pocket on ErbB-2 since this region internalizes trastuzumab upon its binding (Austin et al., 2004) in a Caveolae/Lipid-Raft Mediated mechanism (Liang et al., 2021). The last frame of each MD trajectory entered the docking step. We used HADDOCK and PRODIGY servers to find best potential binding modes and ΔG for each CP, respectively. The trastuzumab-binding pocket of ErbB-2 includes its domain IV residues Pro579, Glu580, Asp582, Gln583, Lys591, Asp592, Pro593, Pro594, Phe595, Asp607, Leu608, Tyr610, Lys615, Gln624, Cys626, and Pro627 (Figure 6). The results indicated that except CP8, other CPs showed higher docking scores compared to the well-known anti-cancer agent, trastuzumab (with the binding score of −90.), suggesting that they may have lower energy constraints for binding to the receptor (Table 3). Regarding the binding affinity (ΔG), CPs 2 and 10 had the maximum quantities suggesting their highest potential for binding (Table 3). This can be supported by the highest numbers of hydrogen bonds these CPs established (15 and 10 hydrogen bonds, respectively; Figures 7C, D). Moreover, it can be seen that CPs 2 and 10 had higher binding affinity compared with trastuzumab (ΔG = −10.3 (kcal.mol−^1^)).

**TABLE 3:** Docking scores and ΔG of CPs obtained from the HADDOCK and PRODIGY servers.

Linker	Peptide	CP	Docking score	ΔG (kcal.mol−^1^)
EAAAK	MARAKE	2	−111.6 ± 14.4	−12.6
	WYAWML	5	−116.4 ± 4.4	−8.5
GGGGS	KCCYSL	7	−96.6 ± 5.1	−9.6
	MARAKE	8	−74.1 ± 3.6	−8.9
	MSRTMS	10	−110.0 ± 10.2	−13.3

## 4 Conclusion

Our research aimed to design novel anticancer chimeric suppressors of protein synthesis in ErbB2 -positive cancer cells by ricin, a natural toxic. To overcome the non-specific toxicity of ricin, we used the catalytic chain A (ricin_A_) whose specific recognition potential against the ErbB2 receptor was enhanced by adding specific peptides having an affinity for the ligand-uptaking domain of the receptor. Our computational studies suggest CP2, and 10 as potent ribosome inactivating candidates due to their maintained natural conformation of ricin_A_ and having favorable affinity against ErbB2. Although the present computational study provides two potential candidates for ErbB-2 amplified cancers, an experimental process needs to be established, a matter which is the theme of our future study.

## Data Availability

The original contributions presented in the study are included in the article/Supplementary Material, further inquiries can be directed to the corresponding author.
