# Supplementary material for: Computational design of fusion proteins against ErbB2-amplified tumors inspired by ricin toxin
Source: Front Mol Biosci. 2023 Mar 2;10:1098365. doi: 10.3389/fmolb.2023.1098365 (PMC10018397; doi:10.3389/fmolb.2023.1098365)
Supplement: Supplementary file 1 [file Table1.DOCX]

| **Ricin chain A** | **1** |
| --- | --- |
| **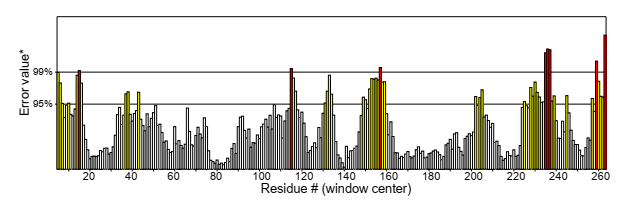** | **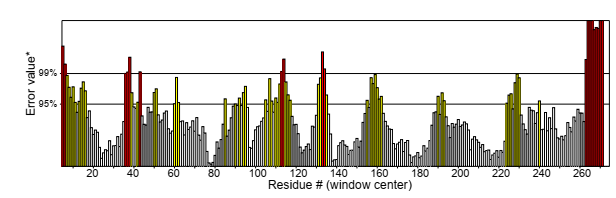** |
| **2** | **3** |
| **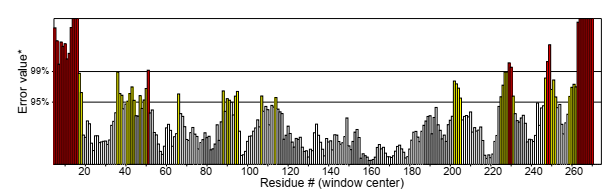** | **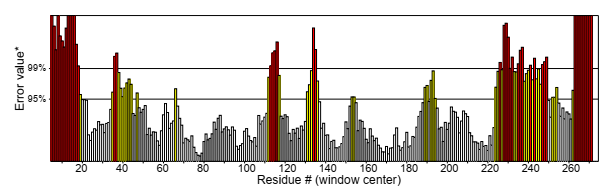** |
| **4** | **5** |
| **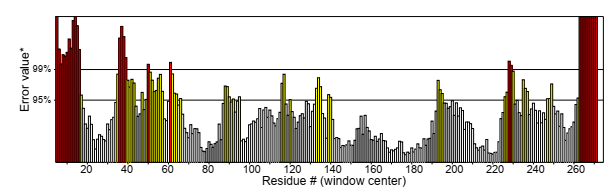** | **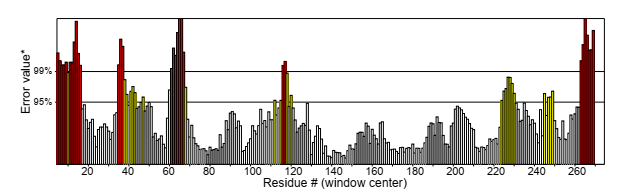** |
| **6** | **7** |
| **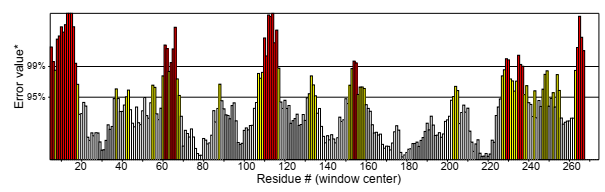** | **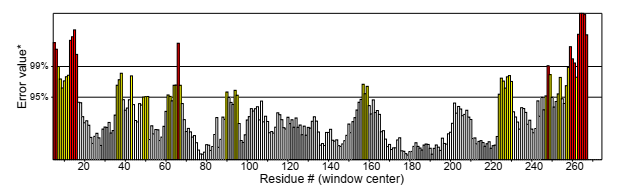** |
| **8** | **9** |
| **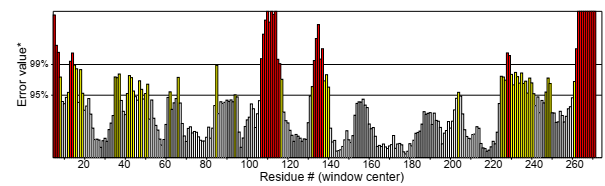** | **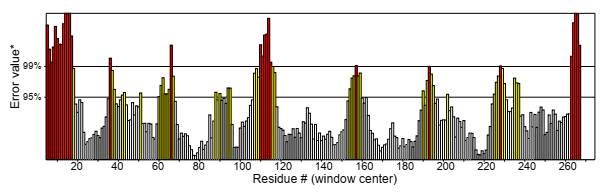** |
| **10** | **11** |
| **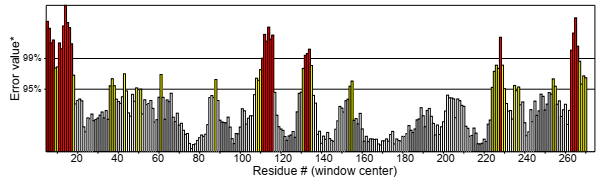** | **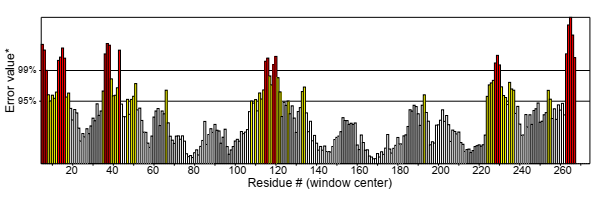** |
| **12** |  |
|  |  |
| **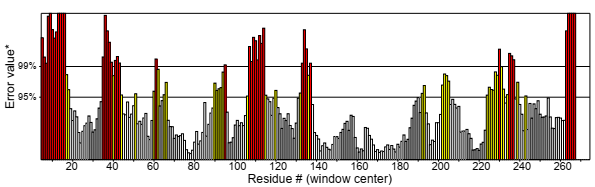** |  |
| **Figure S1- the results of verifying the homology modeled structures by ERRAT server.** | |

| **Ricin chain A** | **1** |
| --- | --- |
| **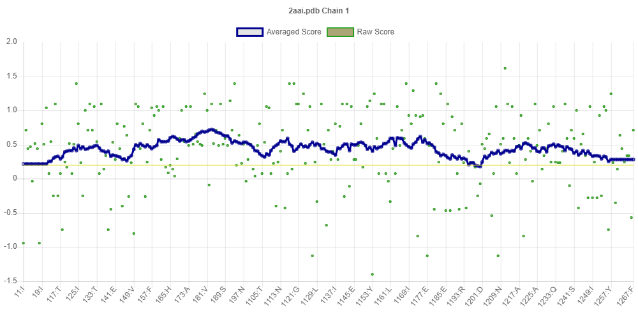** | **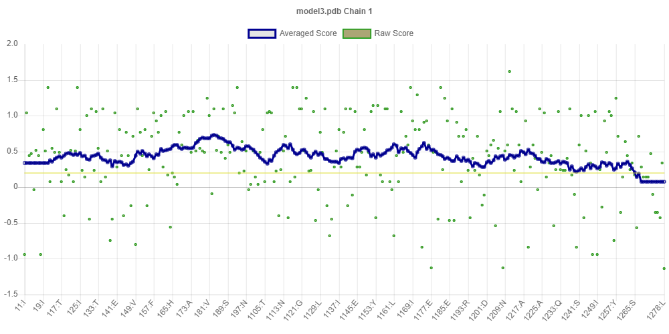** |
| **2** | **3** |
| **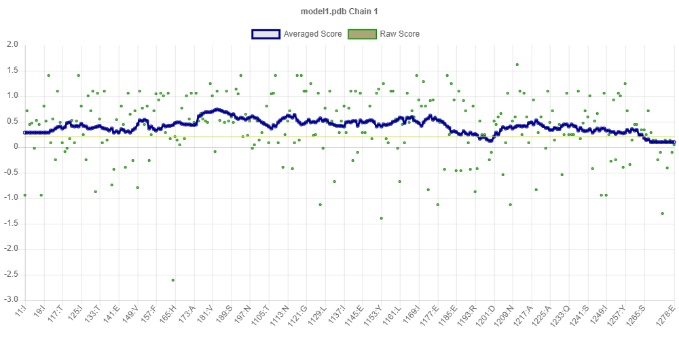** | **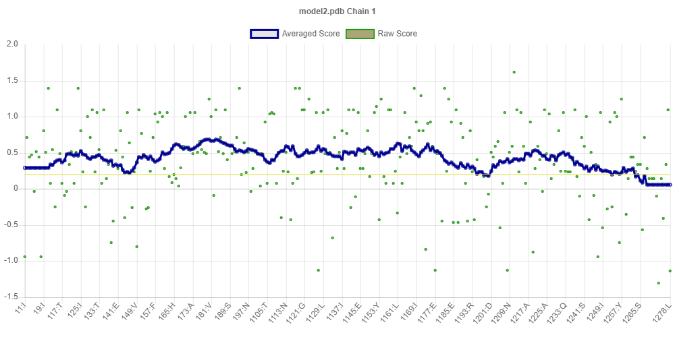** |
| **4** | **5** |
| **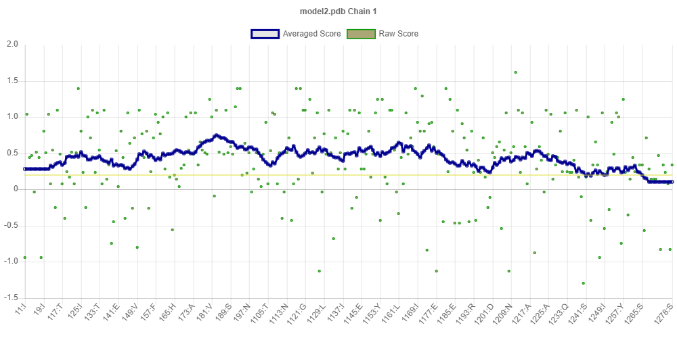** | **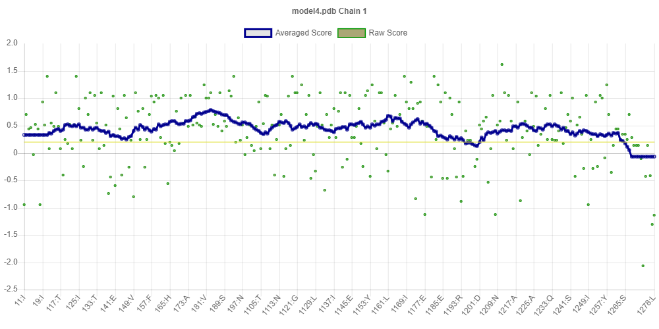** |
| **6** | **7** |
| **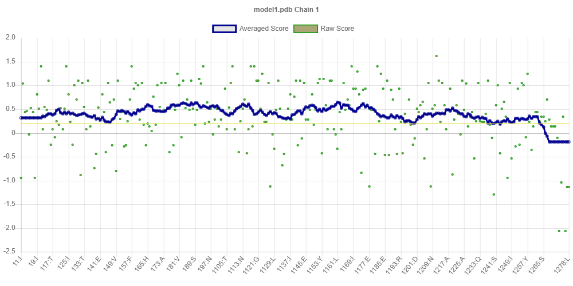** | **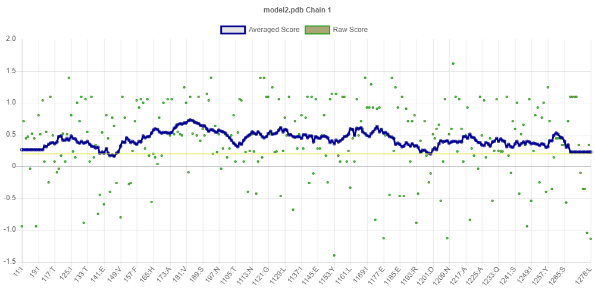** |
| **8** | **9** |
| **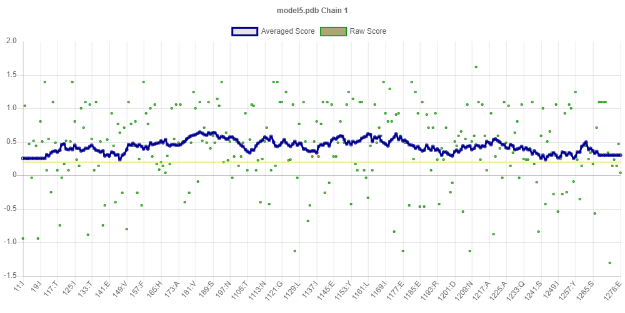** | **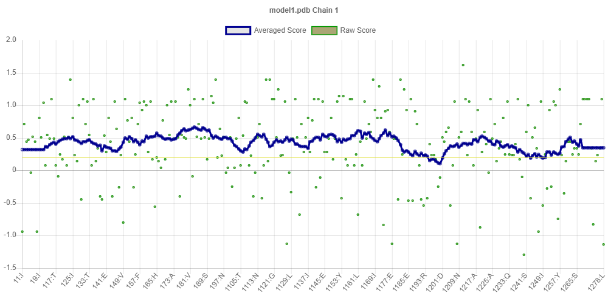** |
| **10** | **11** |
| **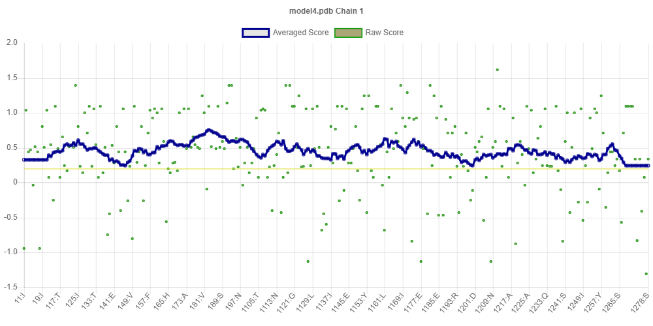** | **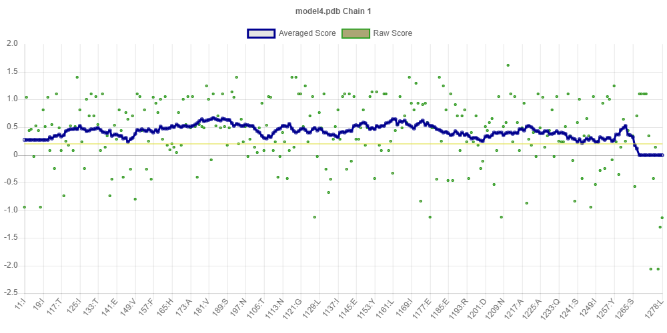** |
| **12** |  |
| **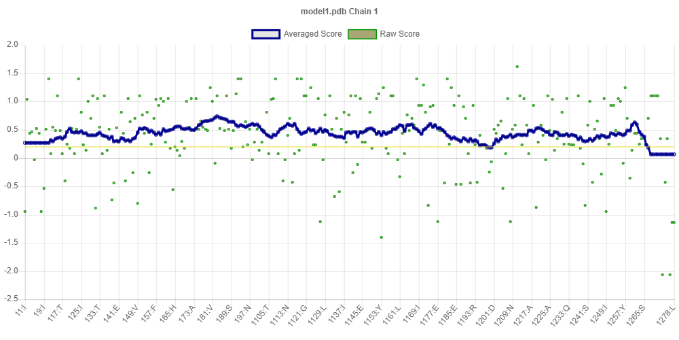** |  |
| **Figure S2- the results of verifying the homology modelled structures by Verify 3D server.** | |

| **Ricin chain A** | **1** | **2** |
| --- | --- | --- |
| **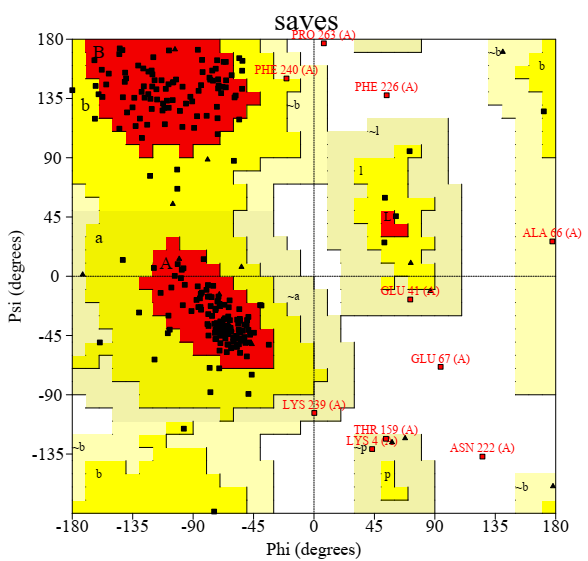** | **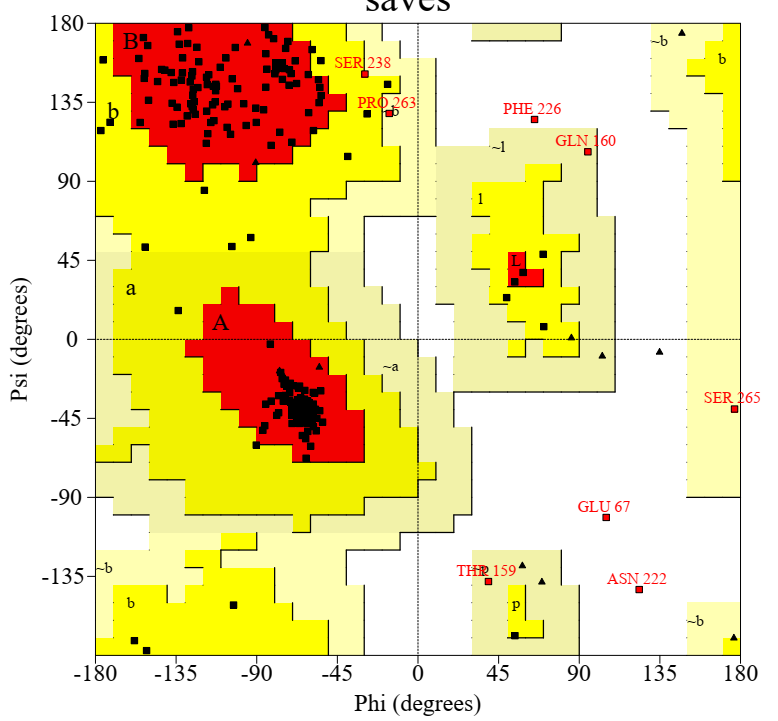** | **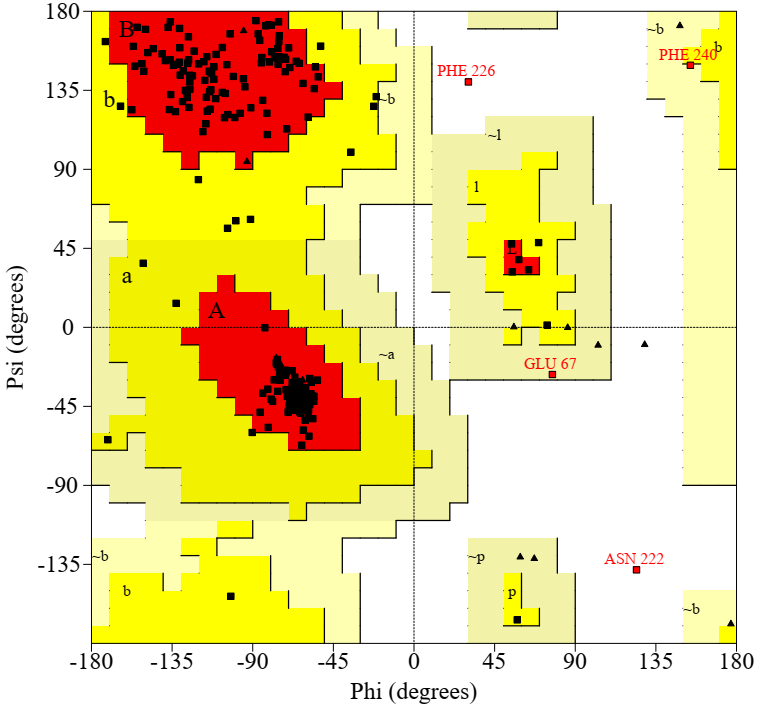** |
| **3** | **4** | **5** |
| **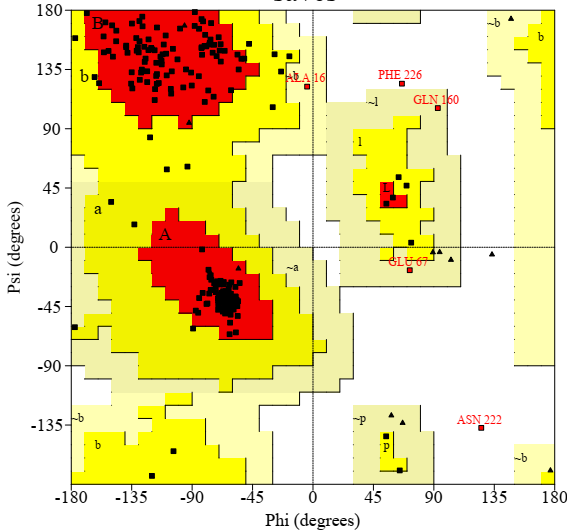** | **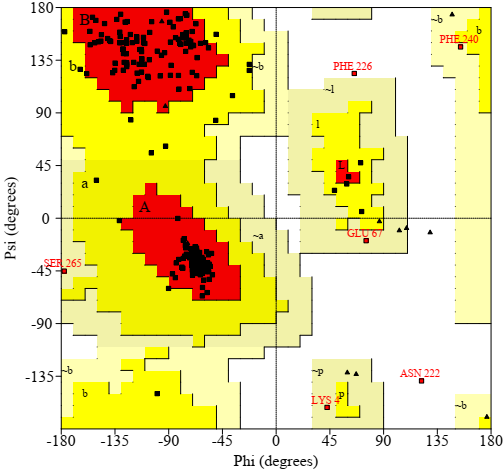** | **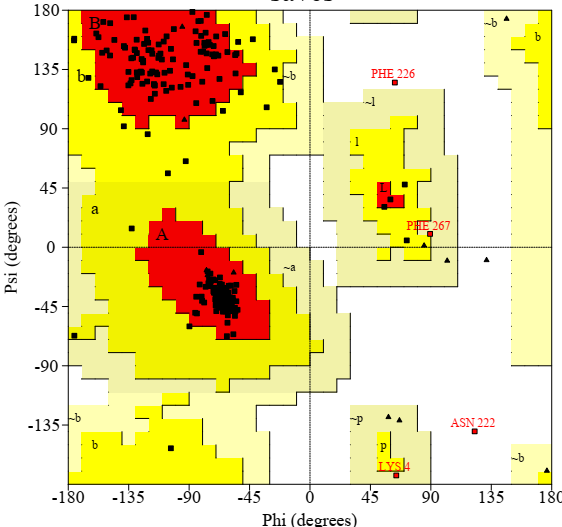** |
| **6** | **7** | **8** |
| **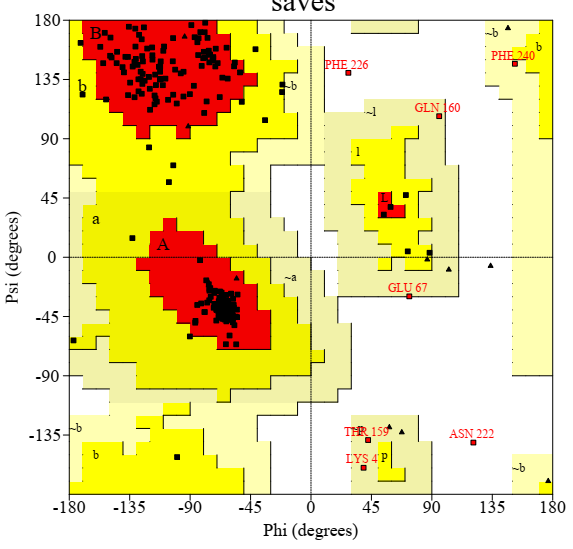** | **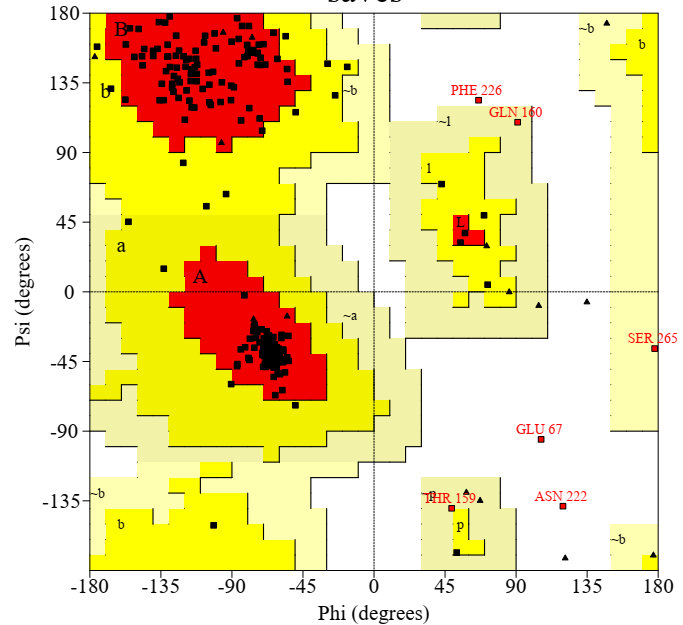** | **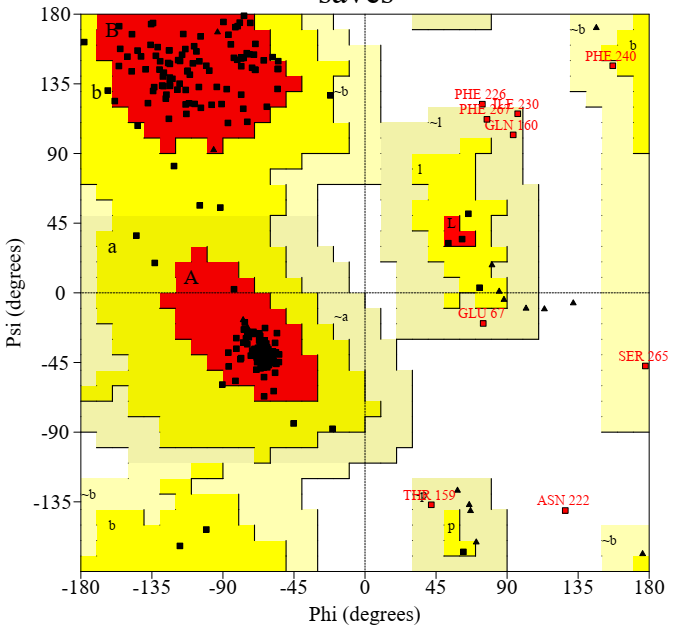** |
| **9** | **10** | **11** |
| **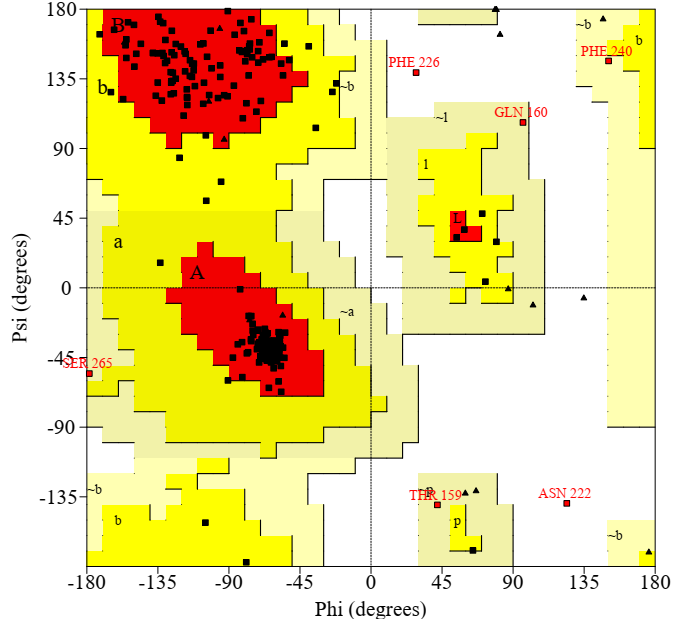** | **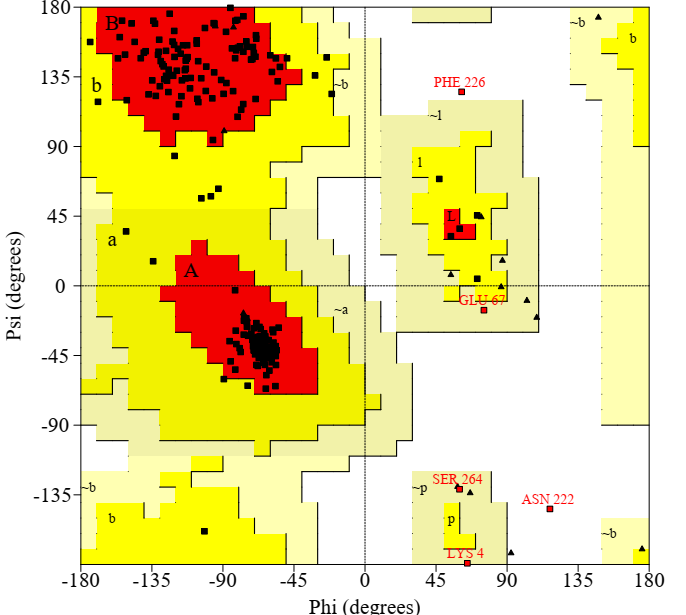** | **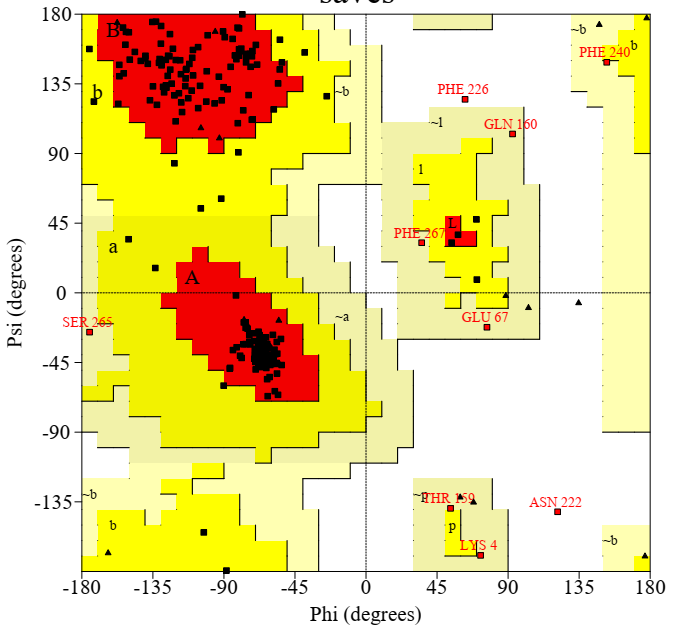** |
| **12** |  |  |
| **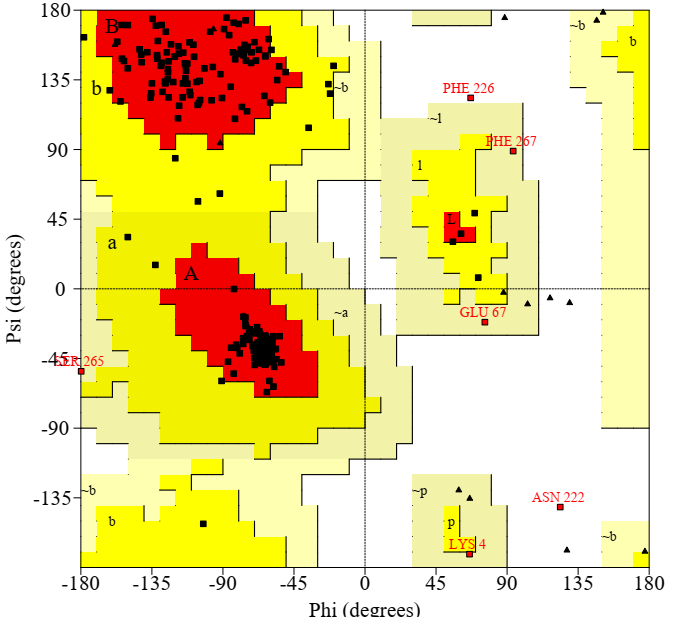** |  |  |
| **Figure S3- Ramachandran plots of models of ricin chain A, and chimeric structures CPs 1-12 by PROCHECK server** | | |

| **Table S1- Toxicity assessment of CPs** | | | |
| --- | --- | --- | --- |
| ID | ToxDL  Score | Result | Motif assessment |
| CP1 | 9.11 | Non-toxic | 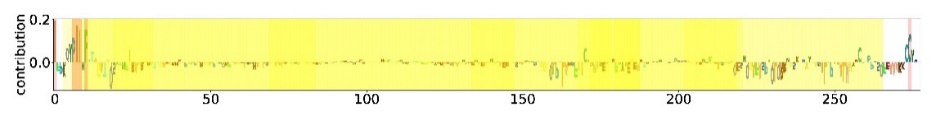 |
| CP2 | 3.05 | Non-toxic | 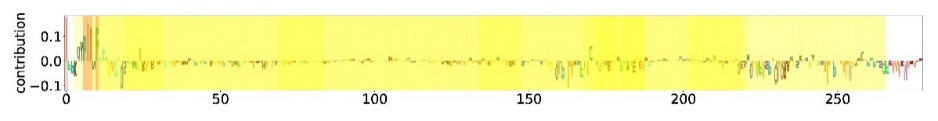 |
| CP3 | 3.14 | Non-toxic | 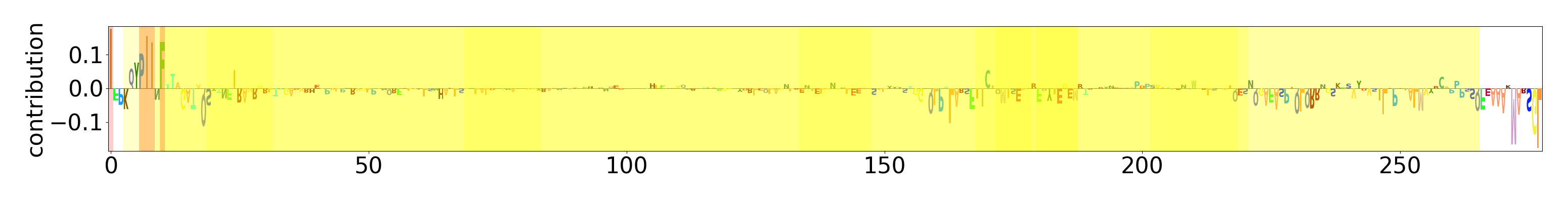 |
| CP4 | 2.7 | Non-toxic | 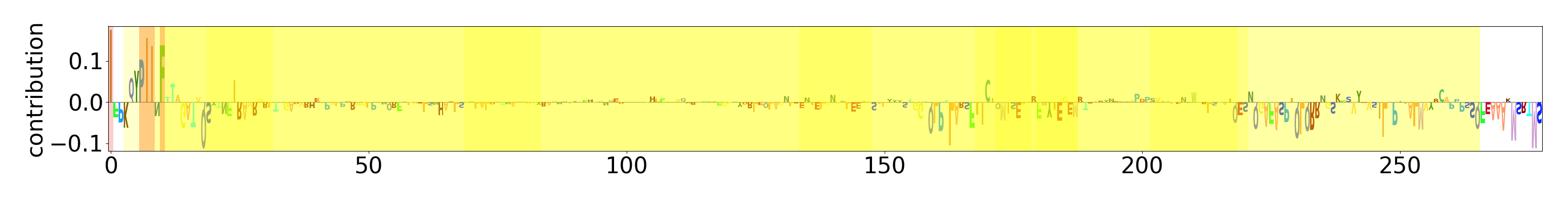 |
| CP5 | 1.26 | Non-toxic | 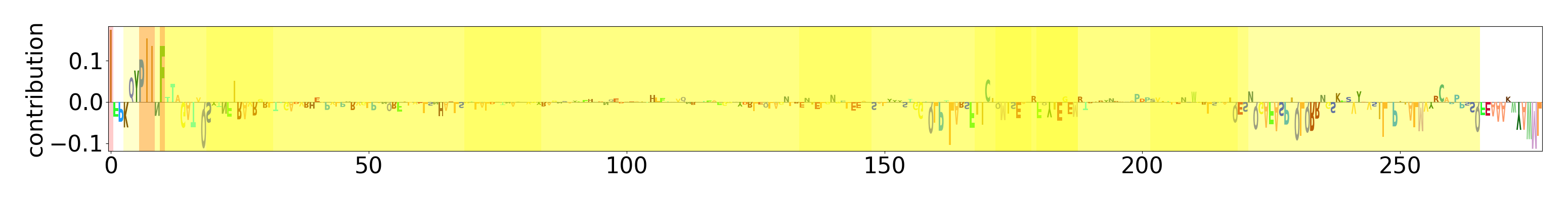 |
| CP6 | 1.7 | Non-toxic | 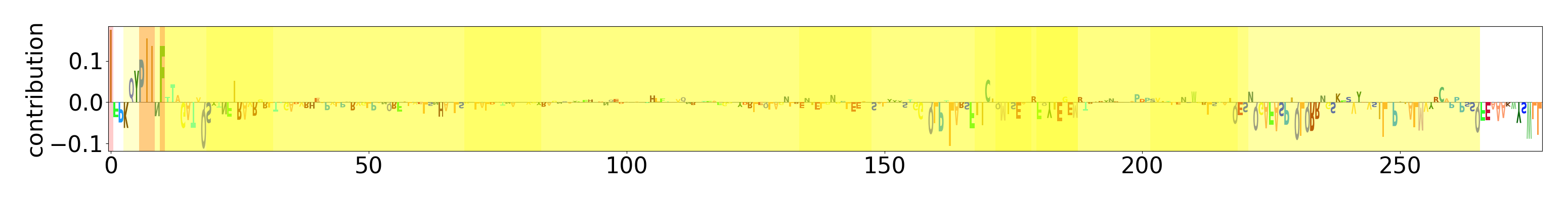 |
| CP7 | 0.00 | Non-toxic | 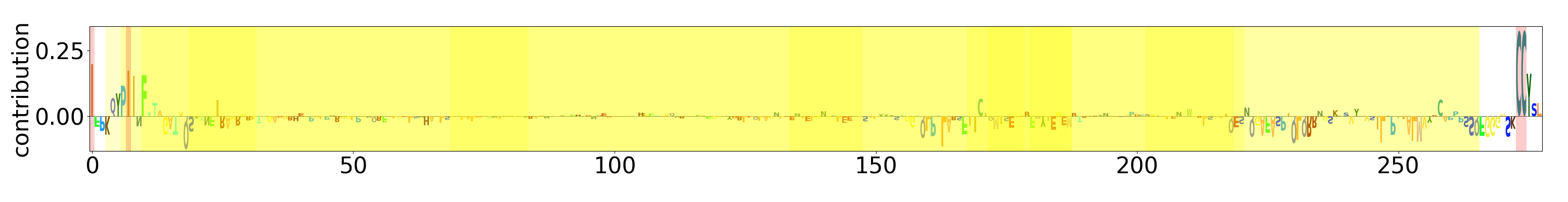 |
| CP8 | 3.31 | Non-toxic | 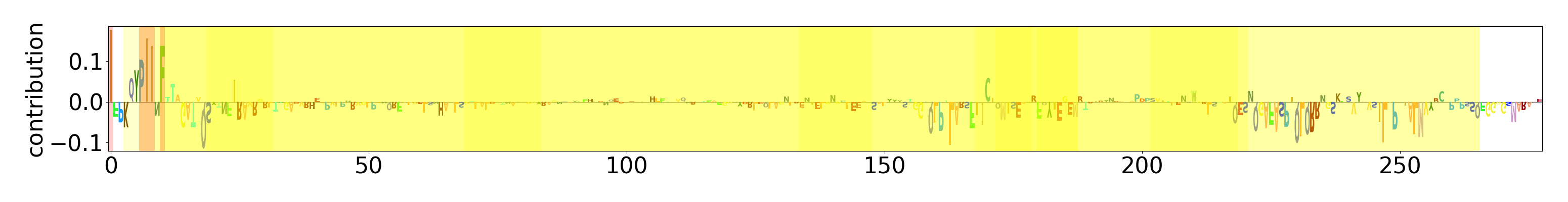 |
| CP9 | 3.33 | Non-toxic | 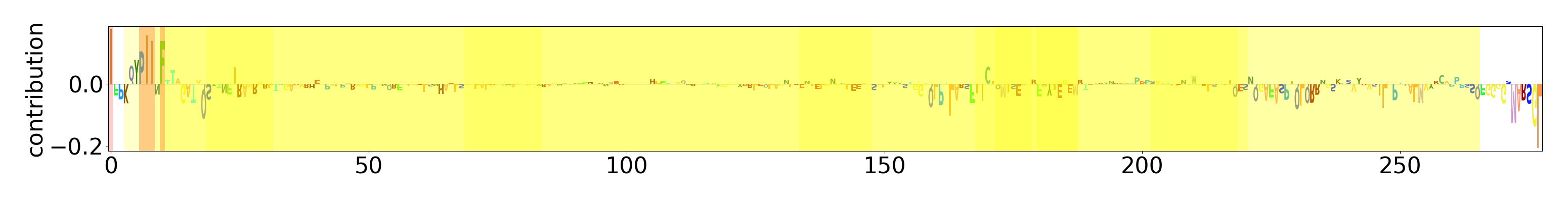 |
| CP10 | 2.31 | Non-toxic | 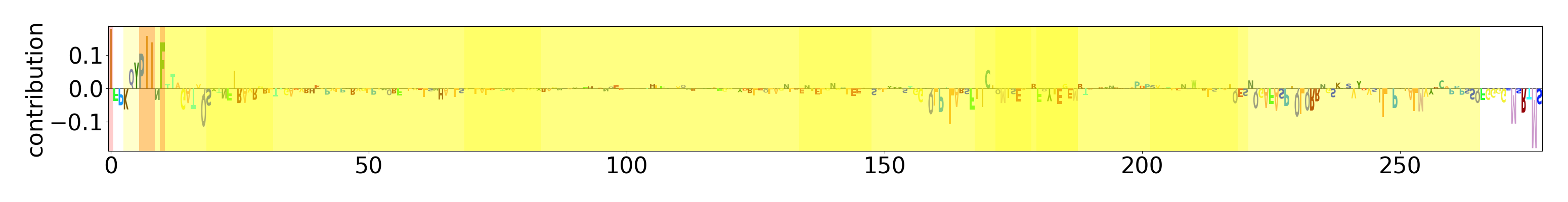 |
| CP11 | 1.8 | Non-toxic | 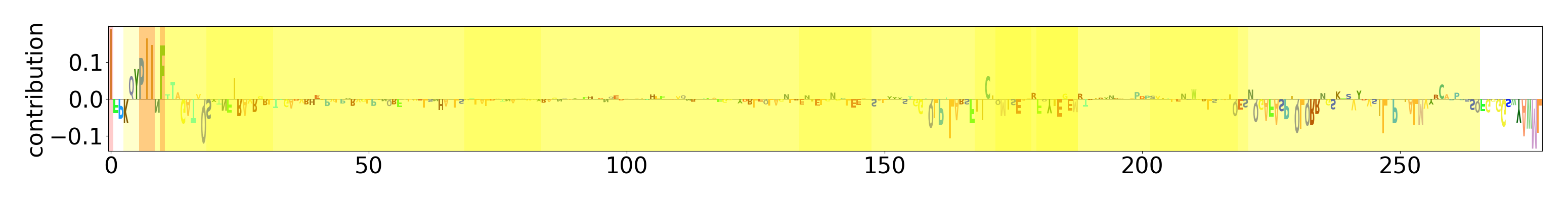 |
| CP12 | 1.6 | Non-toxic | 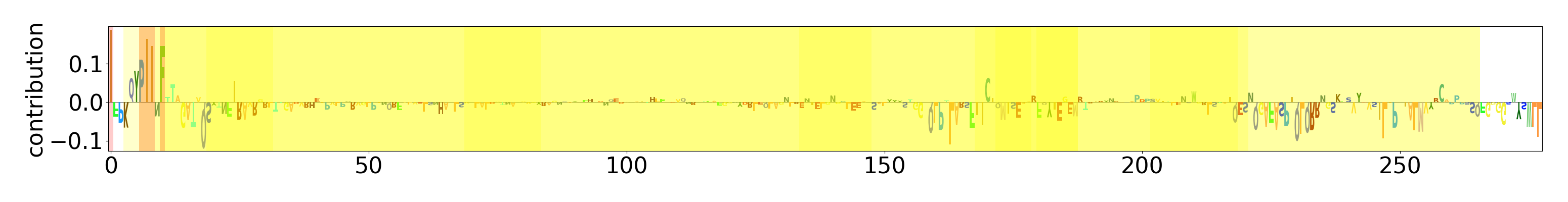 |
